# Editorial for the Special Issue “Functionalized Gels for Environmental Applications 2nd Edition”

**DOI:** 10.3390/gels11060444

**Published:** 2025-06-10

**Authors:** Luca Burratti, Iole Venditti, Paolo Prosposito

**Affiliations:** 1 Istituto Nazionale di Fisica Nucleare, Sezione di Roma Tor Vergata, 00133 Rome, Italy; 2 Sciences Department, Roma Tre University, Via della Vasca Navale 79, 00146 Rome, Italy; iole.venditti@uniroma3.it; 3 Department of Industrial Engineering, University of Rome Tor Vergata, Via del Politecnico 1, 00133 Rome, Italy; paolo.prosposito@uniroma2.it

Dear readers, the second edition of the Special Issue entitled “Functionalized Gels for Environmental Applications” in the journal *Gels* was also a great success. The participation from the scientific community was high, indicating that the interest in this topic is strong. This volume contains ten original manuscripts and one review. Most of the articles are focused on strategies for the purification of water contaminated by different pollutants; certainly, given that water is one of the most precious resources, it is essential to find methods for maintaining its high quality by avoiding dangerous effects not only on human health but also on the entire ecosystem.

Two interesting manuscripts discussed strategies to improve the mechanical properties of hydrogels and their potential use as adsorbent materials for the removal of water pollutants. Specifically, in the research conducted by T. Kawate et al. (Contribution 1), a hydrogel based on interpolyelectrolyte complexes (IPECs) between carboxymethyl cellulose nanofibers (CMCNFs) and chitosan (CS) was prepared by electrostatic crosslinking and compared with the hydrogels of carboxymethyl cellulose (CMC) and CS biopolymers. The presence of the rigid CMCNF altered the mechanism of the IPEC assembly and drastically affected the structure of IPEC hydrogels, donating a higher storage modulus of the CMCNF-CS hydrogel, reaching 13.3 kPa compared with only 3.5 kPa measured for the CMC-CS hydrogel. The adsorption capabilities of CMCNF-CS compared with CMC-CS toward Cu(II), Cd(II), and Hg(II) ions showed a slightly higher adsorption capacity of CMCNF-CS for Cu(II) but similar adsorption capacities for Cd(II) and Hg(II). The authors claimed that although the absorption capacity does not change by inserting cellulose nanofibers, a higher stiffness and a frequency-independent loss modulus bring benefits in practical applications that require stable performance under various dynamic conditions. In the work conducted by L. M. Araque et al. (Contribution 2), the mechanical properties of hydrogels based on linear polyethyleneimine (PEI) chemically crosslinked with ethyleneglycoldiglycidyl ether (EGDE) were improved by ionic crosslinking with sodium tripolyphosphate (TPP). The addition of small amounts of TPP, 0.03 to 0.26 mmoles of TPP per gram of material, to the PEI-EGDE hydrogel increased the deformation resistance from 320% to 1080%, respectively. The two hydrogels (PEI-EGDE and PEI-EGDE-TPP) were tested as adsorbent materials for different water pollutants such as penicillin V, methyl orange (MO), and copper(II) ions. Regarding penicillin V and Cu(II), the adsorption capabilities for both hydrogels showed the same results, while for methyl orange, PEI-EDGE adsorbed more than TPP-modified hydrogel. This can be due to two factors: one related to the lower swelling capacity and a less accessible structure in comparison with the PEI-EGDE hydrogel, while the second factor is that TPP has a net negative charge, which generates electrostatic repulsion with MO molecules.

Different hydrogels for heavy metal ions adsorption have been developed by different research groups. A new method for recycling polyethylene terephthalate (PET) has been presented (Contribution 3) based on microwave-assisted depolymerization of waste PET plastic using polyamine to prepare short aminophthalamide oligomers followed by chemically crosslinking into a hydrogel material. This crosslinked hydrogel showed a high efficiency for removing Cu(II) ions from water. The abundant presence of NH_2_ ligand groups in the hydrogel determines superior adsorption characteristics of the hydrogel that was demonstrated for Cu(II) but potentially applicable to a broad range of other transition metal ions. The proposed hydrogel adsorbent is a sustainable material that simultaneously addresses two environmental issues: waste utilization and pollutant removal. An eco-friendly modified sodium alginate was prepared via one-pot polymerization with acrylic acid by T. Wu and coworkers (Contribution 4); the material was employed in tests for the adsorption of metal ions such as Cu(II), Zn(II) and Ni(II), finding maximum capacities of 367.64, 398.4, and 409.83 mg/g, respectively. In addition, reusability tests were carried out for ten cycles, and the effective metal desorption and adsorbent recovery from acidic solutions was observed to be more than half of the original adsorption capacity. F. Chen and collaborators (Contribution 5) developed water-insoluble poly(gamma-glutamic acid)-based hydrogels as heavy metal adsorbents. The prepared hydrogels exhibited good adsorption capabilities for removing heavy metal ions such as Cu(II), Cr(VI), and Zn(II). The adsorption isotherm studies revealed that the experimental points follow the Langmuir model for all metal ions, obtaining maximum adsorption values equal to 21 mg/g, 212 mg/g, and 198 mg/g for Cu(II), Zn(II), and Cr(VI), respectively. In addition, reusability tests of the hydrogels were conducted, and a decrease of only 22% of the adsorption efficiency for the fifth cycle compared with the first cycle was observed.

Other gel systems have been developed, for instance, in the agriculture sector. X. Tao et al. (Contribution 6) designed a strategy to synthesize a superabsorbent gel from corn straw by exploiting gamma rays; the resulting material exhibited a maximum absorption of water equal to 1033 g for one gram of gel and 90 g/g in a solution of 0.9% in wt. of NaCl. This gel can be used in agriculture as a fertilizer due to the presence of urea inside the material. In contrast, X. Hu et al. (Contribution 7) demonstrated that their composite gel (corn stalk biochar/sludge biochar–sodium alginate gel-hydroxyapatite): CB/SB-SA-HAP can effectively decrease soil pH after a treatment of 21 days, reaching a value of 7. The soil treated with this gel increased the availability of different important components, such as alkali-hydrolysable nitrogen (34.89~57.91%), available phosphorus (35.93~56.55%), and available potassium (36.41~56.80%), improving the plant growth.

Moreover, the system conceived by B. Jiang and colleagues (Contribution 8) is based on a Cu_2_O–SiO_2_–acrylic resin primer for anchoring and controlling copper ion release with a dissipative double-network double-anchored hydrogel that boosts the mechanical strength and anti-biofouling performance in a marine environment. The mechanism of resisting biological adherence is mainly due to the surface of the hydrogel that is not fit for microorganisms to live on, and once a microorganism adhered to it accidentally, the Cu(II) ions in the gel would kill it immediately.

A gel designed by V. Kyselová and coworkers (Contribution 9) based on silica gel impregnated with polyethyleneimine was employed in carbon dioxide sequestration. This modified system was compared with non-impregnated silica gel in terms of chemisorption and physisorption of carbon dioxide, finding that in the range from 30 °C (10.26 g/100 g of material) to 100 °C (8.13 g/100 g of material), the impregnated silica gels were able to adsorb almost constant CO_2_. Conversely, the pristine silica gel had a drop in the sorption quantity, passing from 30 °C (7.44 g/100 g of material) to 100 °C (0.68 g/100 g of material).

Photocatalysis is one of the effective ways to degrade organic pollutants in water. In the research of X. C. Yang et al. (Contribution 10), a ternary aerogel was developed: agar was used as the adsorption module to provide abundant pore structure, carbon dots (CDs) were selected as light energy conversion components, and graphitic carbon nitride (g-C_3_N_4_) was used as the main material of the catalyst (Agar/CDs/g-C_3_N_4_). The system was able to degrade to an almost fully amoxicillin antibiotic under a xenon lamp (visible light source) in only 45 minutes. This result was mainly due to the wide visible light utilization range and high carrier separation efficiency of the system and to the aerogel porosity, which can provide more active sites for redox reaction in photocatalytic activity.

Finally, the interesting review by F.R. Amalia and coworkers focused on photocatalysis approaches based on gels for different environmental purposes, such as solar-energy-based reactions, water treatment, photodynamic cancer therapies, and fundamental research. In addition, the review sheds light on critical aspects of these photocatalytic materials, for instance, recyclability and stability. The authors concluded by claiming that gel photocatalysts are highly promising materials for broad environmental applications when the stability issue is successfully solved (Contribution 11).

In conclusion, the articles in this volume are of high quality, and as Guest Editors, we are satisfied with how innovative compounds and advanced tools are presented, which are rapidly evolving in the field of multidisciplinary research related to this topic. We are confident that this collection will contribute to this research area, whose interest is gradually growing over time, providing our readers with a broad and updated scenario of the situation.

